# Structural analysis of PIM1 kinase complexes with ATP-competitive inhibitors

**DOI:** 10.1038/s41598-017-13557-z

**Published:** 2017-10-17

**Authors:** Jozefina Bogusz, Karol Zrubek, Krzysztof P. Rembacz, Przemyslaw Grudnik, Przemyslaw Golik, Malgorzata Romanowska, Benedykt Wladyka, Grzegorz Dubin

**Affiliations:** 1 Malopolska Centre of Biotechnology, Jagiellonian University, Gronostajowa 7a, 30-387 Krakow, Poland; 2 Faculty of Biochemistry, Biophysics and Biotechnology, Jagiellonian University, Gronostajowa 7, 30-387 Krakow, Poland

## Abstract

PIM1 is an oncogenic kinase overexpressed in a number of cancers where it correlates with poor prognosis. Several studies demonstrated that inhibition of PIM1 activity is an attractive strategy in fighting overexpressing cancers, while distinct structural features of ATP binding pocket make PIM1 an inviting target for the design of selective inhibitors. To facilitate development of specific PIM1 inhibitors, in this study we report three crystal structures of ATP-competitive inhibitors at the ATP binding pocket of PIM1. Two of the reported structures (CX-4945 and Ro-3306) explain the off-target effect on PIM1 of respectively casein kinase 2 and cyclin-dependent kinase 1 dedicated inhibitors. In turn, the structure with CX-6258 demonstrates a binding mode of a potent, selective inhibitor of PIM1, PIM2, PIM3 and Flt-3 kinases. The consequences of our findings for future inhibitor development are discussed.

## Introduction

PIM1 kinase (Provirus Integration site for Moloney leukemia virus) is a member of constitutively active calcium/calmodulin-regulated serine/threonine kinase family. Under physiological conditions PIM1 is constitutively expressed at low level in a number of tissues^1,2^. Its level significantly increases in response to various growth factors, mitogens and cytokines, as it is regulated by JAK-STAT and NF-κB pathways^3^. PIM1 is overexpressed in a wide range of human tumors, both of hematopoietic and epithelial origin, including myeloid and lymphoid acute leukemia, diffuse large cell lymphoma (DLCL) and neoplastic prostate cancer, suggesting its involvement in oncogenic processes^4^. Additionally, in hematopoietic malignancies, PIM1 expression correlates with poor prognosis.

Oncogenic activity of *Pim1* has been established in transgenic mice carrying *Pim1* with upstream immunoglobulin enhancer sequences (Eµ), resulting in increased Pim1 expression in T cells. These mice developed spontaneous T cell lymphomas, albeit with long latency. The oncogenic capacity of Pim1 kinase dramatically increased with higher expression levels. *Pim-1* transgenic mice carrying two copies of the transgene or a single copy of the transgene with optimized ribosomal-binding site succumbed to lymphomas before reaching reproductive age^3^. Pim1 effectively collaborates with MYC in tumorigenesis. Overexpression of MYC induces apoptosis and overexpression of Pim1 overcomes MYC-induced apoptosis to permit oncogenesis. Pim1 and MYC oncogenic cooperation is one of the strongest described – the transgenic mice which co-express *Eµ-Pim1* and *Eµ-Myc* succumb to lymphomas *in utero* or around birth^3^.

The main mechanisms by which PIM kinases exert their oncogenic activities include: modulation of MYC transcriptional activity (PIMs phosphorylate MYC on S62 and S329, which results in increased MYC protein stability and thereby transcriptional activity), regulation of cap-dependent translation, regulation of cell cycle progression, and pro-survival signaling counteracting the increased sensitivity of tumor cells to apoptosis^3^.

PIM1 has been suggested an attractive drug target in cancer. The following facts make PIM kinases especially attractive for pharmacological inhibition: (i) The phenotype of *Pim1* deficient mice is mild which might point to a favorable toxicity profile for PIM inhibitors. (ii) The proof-of-concept of PIM inhibitor based therapy has been established where *in vivo* growth of xenograft leukemia and adenocarcinoma was reduced by a PIM inhibitor. (iii) The ATP binding mode at the PIM binding site is significantly different compared to the majority of protein kinases. The latter characteristics is especially interesting for drug design, promising the possibility for development of specific inhibitors^3^.

PIM1 adopts a typical bi-lobed kinase fold. The N-terminal domain (residues 37–122) consists mainly of β-strands and a single α-helix. The C-terminal domain (residues 126–305) is primarily α-helical. The domains are connected *via* a hinge region (residues 123–125). The ATP binding site of PIM1 kinase locates between the two domains and is flanked by two loops: a glycine-rich loop (G-loop; residues 44–52) and an activation loop (residues 185–204), and a hinge region^5,6^. The uniqueness of ATP binding in PIM1 manifests in interactions within the hinge region. Most protein kinases interact with ATP by forming two hydrogen bonds within this region, while the hinge region of PIM1 lacks one of the canonical hydrogen bond donors within the backbone instead containing a proline residue (Pro123) at equivalent position^7^. This distinguished feature established the PIM1 protein kinase as an attractive target for drug design.

**Figure 1:**
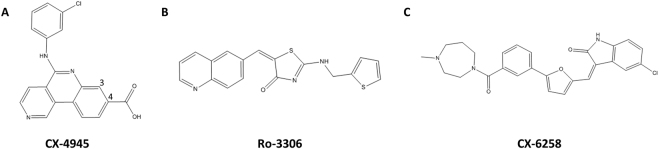
Chemical structures of inhibitors used in the study. (**A**) CX-4945 (**B**) Ro-3306 (**C**) CX-6258.

Binding mode of a number of competitive inhibitors of PIM1 was characterized previously^8^, however, selectivity issues are still not fully resolved at structural level. To facilitate further development of specific inhibitors of PIM1 kinase, here we characterize the binding modes of three structurally distinct inhibitors. CX-4945 (silmitasertib, Fig. 1A) is currently being developed as casein kinase 2 (CK2α) inhibitor in phase I/II clinical trials in leukemia^9^. We selected this inhibitor to reveal the structural basis of its known, albeit relatively weak, off-target effect on PIM1 (IC_50_ = 0,216 µM)^10^. Ro-3306 (Fig. 1B) is a relatively selective inhibitor of cyclin-dependent kinase 1 (CDK1). Its binding mode at the active site of CDK2 was determined previously^11^ and we were interested in providing a structure with an off-target kinase PIM1 for comparative analysis. CX-6258 (Fig. 1C) in turn was selected to provide a binding mode of a potent, selective inhibitor of PIM1, PIM2, PIM3 and Flt-3 kinases. This also allowed experimental evaluation of a CX-6258 interaction model previously proposed by Haddach *et al*.^12^ in the absence of crystal structure of the complex, where the lactam amine and carbonyl were predicted to interact with Lys67 and Asp186 and additional, water mediated interactions were presumably provided by the carboxylate moiety of the inhibitor and the main chain of Asp128 and Glu171.

## Materials and Methods

### *PIM1* expression and purification

A truncated variant of *PIM1* (residues 29–313) was cloned into pETDuet-1 vector (Novagen) to obtain N-terminally His-tagged PIM1 expression construct. His-PIM1 was overexpressed in *Escherichia coli* Rosetta strain in LB medium supplemented with ampicillin and chloramphenicol at 22 °C. 20 hours after induction with 0,5 mM isopropyl β-D-1-thiogalactopyranoside (IPTG; at OD_600_ of 0.5) the cells were harvested by centrifugation, resuspended in 50 mM NaH_2_PO_4_, 300 mM NaCl pH 8.0 containing 10 mM imidazole and lysed by sonication. Lysates were clarified by high-speed centrifugation and the recombinant protein was recovered on Ni-NTA Agarose (GE Healthcare) in lysis buffer. After thorough washing, imidazole gradient was applied and fractions containing PIM1 were pooled and dialyzed against 20 mM Tris, pH 8.0 containing 50 mM NaCl, 5 mM CaCl_2_ and 5 mM β-mercaptoethanol. The protein was further purified using size-exclusion chromatography in crystallization buffer (20 mM Tris pH 8.0 containing 200 mM NaCl, 2 mM CaCl_2_, 2 mM MgCl_2_ and 2.5 mM β-mercaptoethanol).

### PIM1 crystallization

His-PIM1 was concentrated to 8 mg/ml and screening was performed using sitting drop vapor diffusion setup. Several conditions were identified and optimized according to art. The best crystals were obtained at room temperature by mixing 2 µl of protein solution with 1 µl of reservoir solution containing 0.1 M Tris pH 8.0 supplemented with 1.2 potassium sodium tartrate tetrahydrate and grew for approximately one week. CX-4945 (5-((3-chlorophenyl)amino)benzo[c][2,6]naphthyridine-8-carboxylic acid), Ro-3306 (5Z-2-((Thiophen-2-yl)methylamino)-5-((quinolin-6-yl)methylene)thiazol-4(5 H)-one) and CX-6258 (E-5-chloro-3-((5-(3-(4-methyl-1,4-diazepane-1-carbonyl)phenyl)furan-2-yl)methylene)indolin-2-one) were obtained from Biovision, dissolved in dimethyl sulfoxide (DMSO) and soaked into the crystals for 24 hours. The crystals were cryoprotected in 25% glycerol in the mother liquor and cryopreserved in liquid nitrogen.

X-ray diffraction data were collected at BESSY, Berlin, Germany and Diamond Light Source, Oxfordshire, United Kingdom. The data were indexed and integrated using XDS software^13,14^. Following steps were performed with CCP4 software package. The data were scaled with SCALA^15^. Molecular replacement was performed using PHASER^16^ with PDB entry 4ALW^17^ as a search model. The structures were refined in multiple rounds of manual model building in COOT^18^, and restrained refinement using Refmac 5.0^19^. Five percent of the reflections were used for cross-validation analysis and the behavior of R_free_ was utilized to monitor the refinement strategy. Water molecules were added using Coot and manually inspected. To overcome the model bias in the map region describing the inhibitor we have employed an iterative-build OMIT map procedure with simulated annealing (SA) using Phenix^20^. Data collection and refinement statistics are summarized in Table 1. The coordinates and structure factors were deposited in Protein Data Bank with accession numbers 5O11, 5O12 and 5O13 for complexes of PIM1 with CX-4945, Ro-3306 and CX-6258, respectively. Structure analysis was performed and figures were prepared using PyMOL (http://www.pymol.org).

**Table 1:** Data collection and refinement statistics.

	CX-4945	Ro-3306	CX-6258
PDB ID	5O11	5O12	5O13
Wavelength (Å)	0.91841	0.91841	0.97949
Resolution range (Å)	48.03–2.40	42.29–2.40	29.26–2.44
Space group	P6_5_	P6_5_	P6_5_
Unit cell	96.05 96.05 80.30 90.00 90.00 120.00	97.67 97.67 80.58 90.00 90.00 120.00	96.74 96.74 81.76 90.00 90.00 120.00
Total reflections	113006 (16789)	88668 (13325)	82680 (10789)
Unique reflections	16422 (2357)	17210 (2511)	16181 (2307)
Multiplicity	6.9 (7.1)	5.2 (5.3)	5.1 (4.7)
Completeness (%)	99.2 (98.5)	100.0 (100)	99.6 (98.7)
Mean I/sigma (I)	11.9 (3.4)	14.7 (2.1)	38.5 (2.9)
Wilson B-factor	37.1	43.9	38.5
R-merge	0.115 (0.564)	0.076 (0.746)	0.125 (0.960)
R-work	0.1620	0.1731	0.1572
R-free	0.2116	0.2227	0.2140
Number of atoms	2297	2211	2290
Macromolecules	2157	2129	2160
Water	97	53	90
Protein residues	270	266	272
RMS (bonds)	0.0151	0.0158	0.0179
RMS (angles)	1.7598	1.9935	2.0274
Ramachandran favored (%)	96.2	95.38	96.3
Ramachandran outliers (%)	0.76	0.77	1.48
Average B-factor	33.45	40.00	35.20
Macromolecules	32.64	39.57	34.19
Solvent	42.44	49.10	47.26

*Data for the highest resolution shell are shown in parentheses.

## Results

### Overall structural features

All structures determined in this study belonged to P6_5_ space group and contained a single PIM1 molecule in the asymmetric unit. Thereby, the mutual arrangement of the molecules was virtually identical in all the characterized crystals. The crystals diffracted to ~2.4 Å resolution. The refined models span the catalytic domain of PIM1 (residues 34–305). Most of the protein is well defined by electron density save for the residues comprising the glycine-rich loop (G-loop), which was partially disordered in two out of the three reported structures (the loop is ordered in CX-6258 containing structure). Additionally, in Ro-3306 containing structure two residues distant from the active site, Asn82 and Gly83 are not defined by the electron density.

PIM1 adopts a typical bi-lobed kinase fold. The C-terminal lobe (residues 126–305) is largely α-helical whereas the N-terminal lobe (residues 37–122) is composed primarily of β-sheets with a single additional α-helix. Compared to other kinases, where the corresponding region is helical or disordered, the N-terminal domain of PIM1 comprises two additional β-sheets (Gly78-Val86)^7^. The active site is situated between the lobes and enclosed by the hinge region (residues 123–125), the G-loop (residues 44–52) and the activation loop (A-loop; residues 185–204). The A-loop of PIM1 adopts a conformation typical for the active state, a feature independent of phosphorylation as characteristic for constitutively active enzymes.

Additional density in the proximity of Cys161 residue was clearly present which was interpreted as oxidation (CX-6258) or β-mercaptoethanol adduct (CX-4945 and Ro-3306). Moreover, the electron density clearly indicated phosphorylation of Ser261, but this has been previously shown an irrelevant artifact of bacterial expression system which, being located far from the active site, does not influence the protein function^3^.

### Binding of CX-4945 at the ATP pocket of PIM1

CX-4945 is an orally available casein kinase II (CK2α) inhibitor characterized by IC_50_ of 13 nM^21^. It is currently evaluated in Phase I/II clinical trials against cholangiocarcinoma (ClinicalTrials.gov identifier: NCT02128282^22^). Apart from CK2α, CX-4945 effectively inhibits PIM kinases^23^. CK2α and PIM1 share a number of analogies. Both are constitutively active serine/threonine protein kinases and both are involved in cell differentiation, proliferation and survival. Likewise CK2α, PIM1 is overexpressed in prostate cancer, leukemia and lymphomas and promotes oncogenesis by phosphorylation of BAD and collaboration with MYC^23^. As such, PIM inhibition by CX-4945 may provide important synergistic part of its mechanism of action and therefore we were interested in determination of the structural basis of this cross-inhibition.

**Figure 2:**
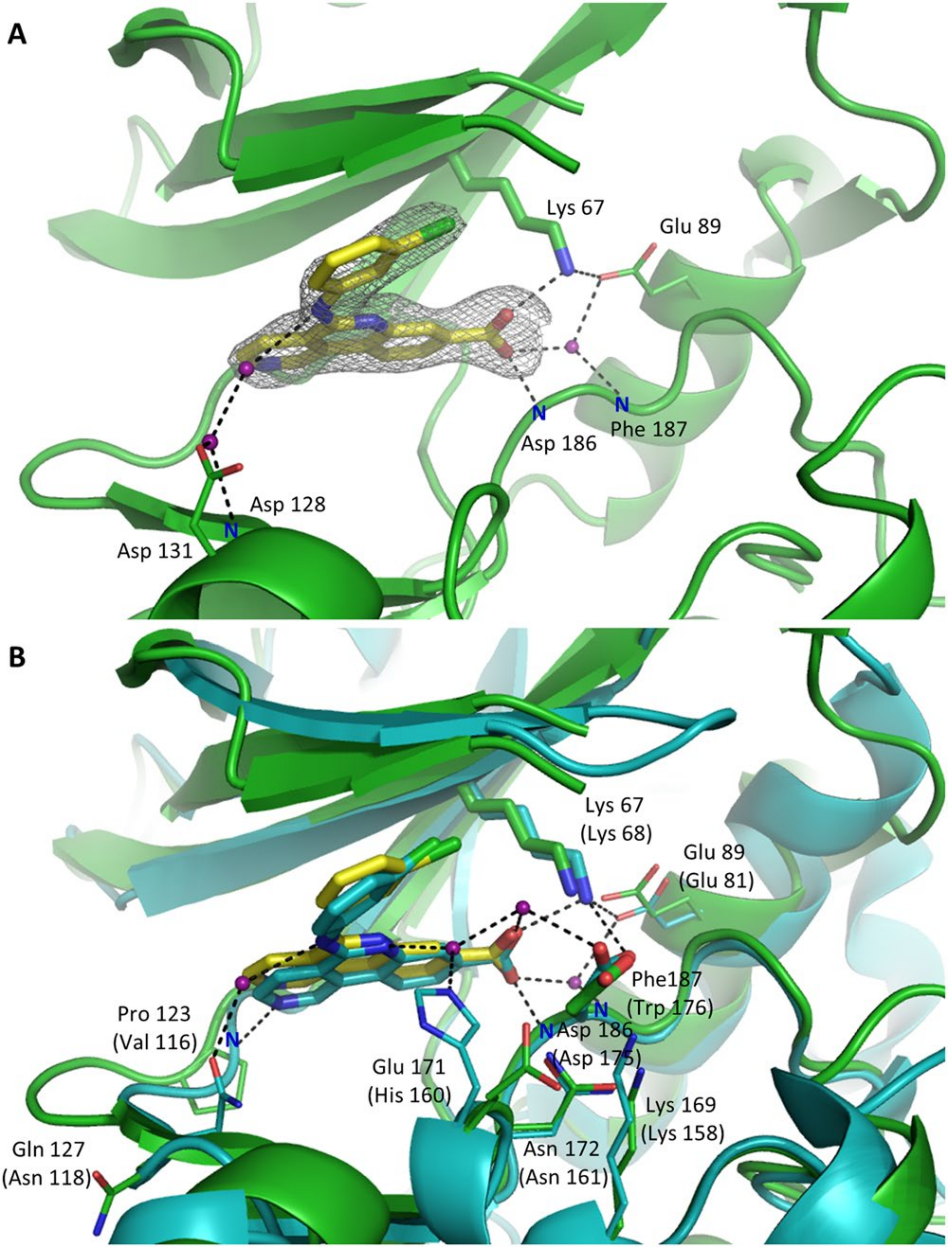
Binding mode of CX-4945 at the active site of PIM1. (**A**) Detailed binding mode and quality of PIM1 co-crystal structure. Electron density defining the inhibitor (SA Fo-Fc omit map contoured at 3σ level) is shown as grey mesh. (**B**) Comparison of the interactions guiding the affinity of CX-4945 at the binding sites of PIM1 (green) and CK2α (blue). Corresponding residues are labeled with PIM1 (on top) and equivalent CK2α residue in parentheses. (**A**,**B**) The protein is shown in cartoon representation and the compound as sticks. Protein residues forming direct and water mediated hydrogen bonds are shown in stick model, thick and thin respectively. Water molecules are depicted as purple spheres. Hydrogen bonds are shown as black dashed lines.

The structure of the complex was solved to 2.4 Å resolution and the electron density clearly defined the inhibitor even before the molecule was introduced into the refinement. CX-4945 is positioned in a pocket formed by a hinge region, glycine-rich loop and the activation loop in an orientation almost identical to that observed in its complex with CK2α kinase (PDB ID: 3PE1^10^, Fig. 2B). In PIM1-CX-4945 complex structure the inhibitor binds the protein through two direct hydrogen bonds (Fig. 2A), while the binding of CK2α involves three direct hydrogen bonds. The first direct hydrogen bond is comparable in both structures - the carboxylate group of CX-4945 interacts with the amino group of Lys67 at the active site of PIM1 and equivalent Lys68 in the structure of a complex with CK2α. Carboxylate moiety was previously used to anchor PIM inhibitors at Lys67. Examples include substituted benzofuran-2-carboxylic acids (5-bromo-1-benzofuran-2-carboxylic acid and 5-bromo-7-methoxy-1-benzofuran-2-carboxylic acid; PDB ID 3R00, 3R01^24^) or VX3 (2,3-diphenyl-1H-indole-7-carboxylic acid; PDB ID 3BGZ^25^) and the interaction of CX-4945 with Lys67 resembles that seen in the above inhibitors.

The second direct hydrogen bond is also comparable in the structures of PIM1 and CK2α. In PIM1-CX-4945 complex the inhibitor’s carboxylate group forms a hydrogen bond with the main chain amide of Asp186 within the A-loop. This residue is equivalent to Asp175 of CK2α, which interacts with the same part of the inhibitor.

The third hydrogen bond stabilizing CX-4945 at the ATP binding site of CK2α is not comparable to the interactions observed in PIM1 structure and involves interaction of a nitrogen within the tricyclic scaffold with the main chain amide group of Val116 residue at the hinge region. Because PIM1 contains Pro123 at an equivalent position, such interaction is not supported.

Additional contacts are mediated through water molecules in both complexes. In CK2α, a water molecule bridges the carboxylic group of the inhibitor and the sidechain of Glu81 and the backbone amide of Trp176, while second water mediates the interaction of the sidechain of Asp175 within the activation loop and the carboxylic group within the inhibitor. Asp186 of PIM1, superimposing with Asp175 of CK2α, does not participate in such water-mediated binding of the inhibitor due to intrinsic interactions with Asn172 (Fig. 2B). Another water molecule connects the inhibitor’s naphthyridine nitrogen with CK2α His160 residue, which adopts an unusual “up” conformation. PIM1 Glu171 residue, which is equivalent to His160 residue of CK2α, points outside the ATP-binding pocket and interacts with Lys169 residue, which might be the reason for lack of interactions of PIM1 with naphthyridine nitrogen of the compound. The fourth water molecule bridges the side chain of CK2α Asn118 and the phenylamino amine group of CX-4945. In PIM1 a single water molecule mediates an interaction of carboxylate substituent of the inhibitor and Phe187 and Glu89, while other water molecules connect the phenylamino amine group of the inhibitor with Asp128 and Asp131 of PIM1.

Binding of the inhibitor at the active site of PIM1 and CK2α is comparable regarding a significant hydrophobic component of the interaction. Within the N-lobe the major contribution is provided by the sidechains of Ala 65 (Val66), Leu 120 (Phe113), Val52 (Val53) and Ile104 (Ile95). Within the C-lobe the majority of the interaction surface is contributed by the sidechains of Leu174 (Met163) and Ile185 (Ile174). The only more significant difference is observed within the hinge region where the sidechain of Val126 within PIM1 provides a hydrophobic contact with the tricyclic scaffold of the inhibitor. This region assumes a different conformation in CK2α and comparable interaction is not present.

Overall, the hydrophobic interactions guiding the recognition of CX-4945 are comparable at the active site of PIM1 and CK2α, whereas certain notable differences are seen in the hydrogen bond network.

### Binding mode of Ro-3306 at the catalytic site of PIM1

A thiazolinone-based Ro-3306 is a reversible, ATP-competitive inhibitor of cyclin-dependent kinase 1 (CDK1), an important regulator of the cell cycle^11^. Ro-3306 has an off-target affinity for PIM1 due to relatively high sequence and structural similarity of the two kinases^26^. We were therefore interested in comparing the binding modes of the inhibitor at the active site of both enzymes to explain, and likely allow to modify the off-target activity in the future.

**Figure 3:**
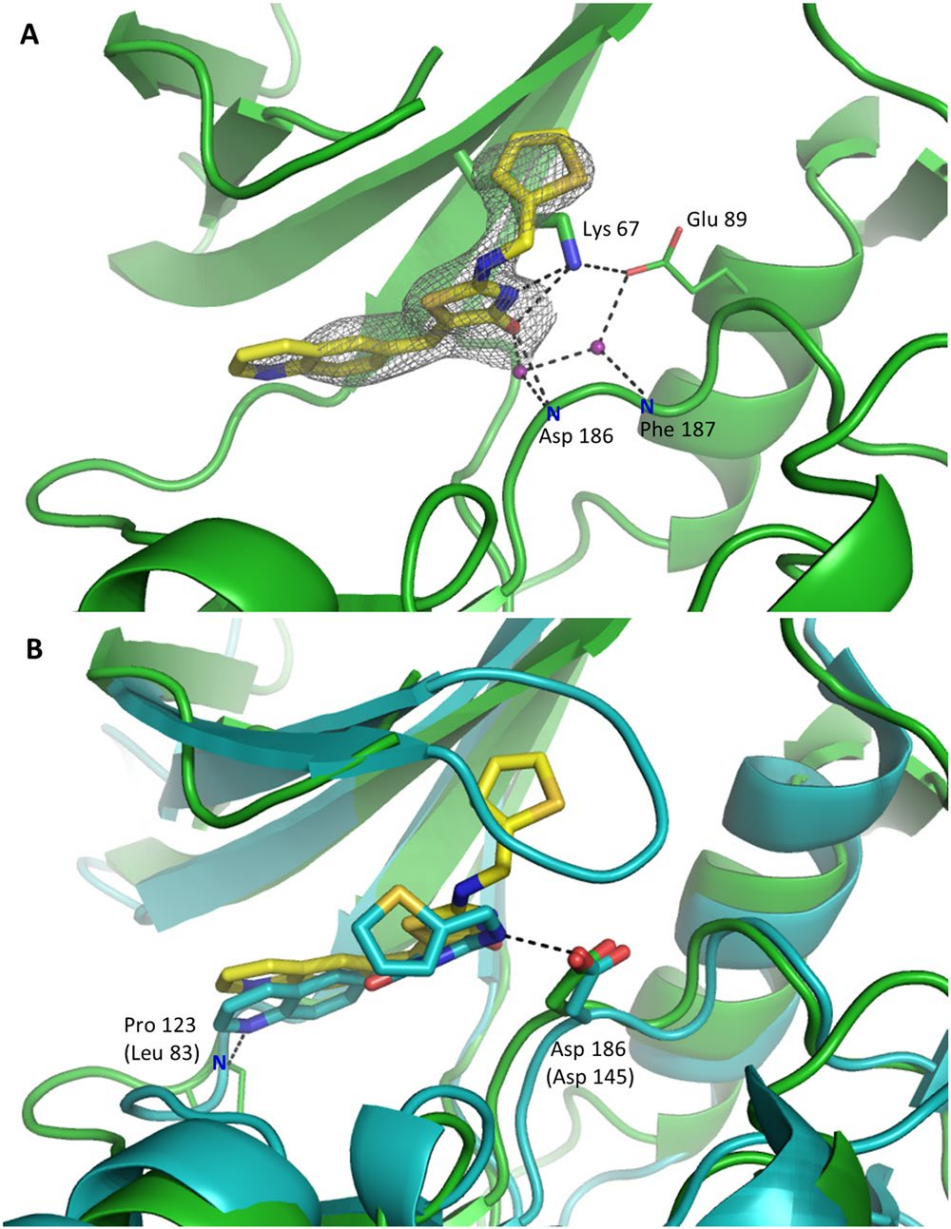
Interactions guiding the recognition of Ro-3306 at the active site of PIM1. Representations, color coding and labeling scheme same as in Fig. 2, save that the bottom panel depicts comparison of the binding mode of Ro-3306 at PIM1 (green) and CDK2 (blue) active sites.

To this end, the structure of PIM1 in complex with Ro-3306 was determined at 2.4 Å resolution. The inhibitor is located at the ATP binding site of the kinase and is well defined by its electron density (Fig. 3A). The quinoline substituent is coplanar to the thiazolone core and the latter contributes two hydrogen bonds with the enzyme. The first hydrogen bond connects the thiazolone nitrogen atom with the sidechain of Lys67. The second bond is bifurcated and connects the oxygen atom with the backbone amide of Asp186 residue, directly or through a water molecule. Additionally, another water molecule bridges the inhibitor and the sidechain of Glu89 and the backbone amide of Phe187. Further, the complex is stabilized by hydrophobic interactions between the thiophene ring and the sidechains of Val52 within glycine-rich loop and Ile185 within activation loop. Additional hydrophobic interactions are contributed by the quinoline moiety and the former two residues and the sidechains of Leu44, Ala65, Ile104, Val126, Leu174. The thiophene moiety is stabilized in a pocket formed by the sidechains of Val52, Lys67, Val69 and the glycine-rich loop which is however poorly defined by electron density in the described region.

Structure of Ro-3306 is only available with CDK2 (K_i_ = 340 nM^27^) and not its primary target kinase CDK1 (K_i_ = 35 nM^27^). Since the CDK2 and CDK1 share significant homology (86% within the active sites), it is of value to compare the interactions guiding the binding of Ro-3306 to CDK2 and to PIM1. The mutual interactions contributed by Ro-3306 and CDK2 (PDB ID 4EOS^11^) and PIM1 are significantly different because CDK2 interacts with different conformational isomer of the inhibitor compared to PIM1. When the structures of PIM1 and CDK2 are overlaid it is immediately visible that thiazolone and thiophene moieties of the inhibitor are differently oriented relative to common structural elements of the compared proteins (Fig. 3B). The thiazolone and quinoline rings are coplanar in PIM1 complex, but not in CDK2 complex. The thiazolone oxygen points towards the A-loop in the former structure, but towards the hinge region in the latter. The largest difference, however, concerns the orientation of the thiophene ring. In PIM1 complex the moiety is directed towards the G-loop, whereas in CDK2 complex it faces the hinge region and fits under the glycine-rich loop which is found in position of the thiophene ring in PIM1 structure. Such orientation of the thiophene ring in CDK2 kinase is stabilized by G-loop which bends towards the active site, a conformation related to phosphorylation of Thr160^28^. Two direct hydrogen bonds mediate the binding of Ro-3306 and CDK2. The first connects the sidechain of Asp145 within the activation loop and the nitrogen within a linker connecting the thiophene and thiazolone rings. In PIM1 complex the inhibitor is anchored at the equivalent Asp186, however using the thiazolone nitrogen and not the linker. The second direct hydrogen bond within CDK2 complex is contributed by the mainchain amide of Leu83 within the hinge region and the quinoline nitrogen. No comparable interaction is observed in PIM1 structure – the quinoline moiety does not contribute any polar interactions with the enzyme. Hydrophobic interactions guiding the binding of the inhibitor at the active sites of both enzymes are comparable in their overall nature, yet differ in certain details. Most significantly, CDK2 interaction is more extended in the hinge region where the sidechains of Phe82 and Leu83 dominate the binding while in PIM1 more significant interactions are contributed by the activation loop where the sidechain of Ile185 provides a larger binding surface compared to equivalent Ala144 in CDK2.

Thiazolone moiety has been previously utilized in PIM1 inhibitor design in substituted benzylidene-1,3-thiazolidine-2,4-diones, TZDs (PDB ID 4DTK^29^). Alignment of PIM1-Ro-3306 complex and that containing TZD demonstrates comparable localization of thiazolone rings within the ATP-binding site of the kinase. In both structures the moiety binds PIM1 through direct hydrogen bonds engaging the sidechain of Lys67 and main chain of Asp186^29^.

### Crystal structure of PIM1 in complex with CX-6258

CX-6258, an oxindole-based compound was recently reported as a potent and selective pan-PIM kinase reversible inhibitor^12,30^ with the reported IC_50_ in the nanomolar range. The compound additionally inhibits the activity of Flt-3 kinase. This could be beneficial since Flt-3 is known to regulate PIM kinase activity in leukemia, albeit the relatively high IC_50_ towards Flt-3 of 0.134 µM^12^ would require further optimization. Preclinical studies indicated that CX-6258 exhibits antiproliferative activity against a panel of cancer cell lines and prevents phosphorylation of BAD and 4E-BP1 in a dose-dependent manner^12^. We were therefore interested in describing its binding mode at the active site of PIM1.

**Figure 4:**
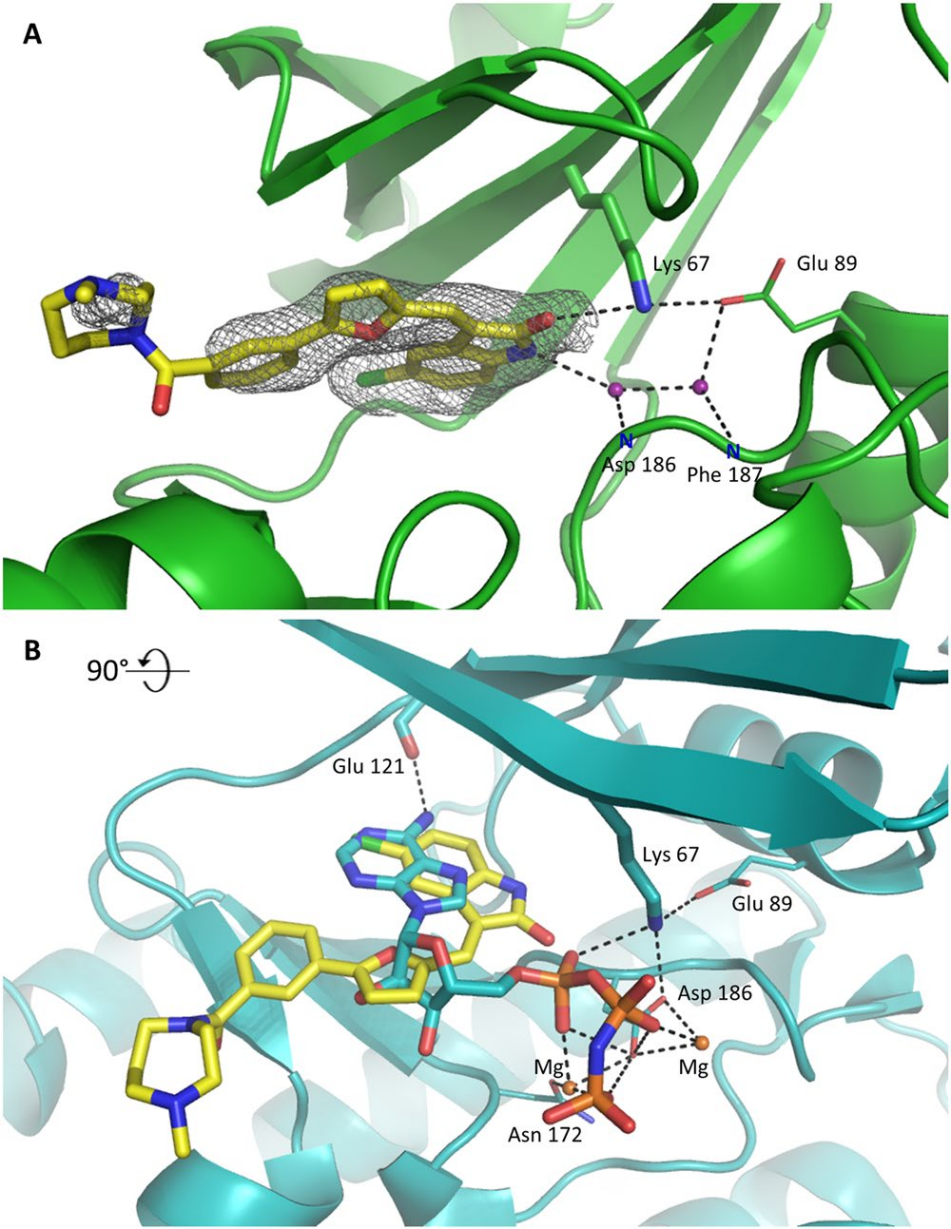
The binding mode of CX-6258 at the active site of PIM1. (**A**) Detailed binding mode and quality of PIM1 co-crystal structure. Electron density defining the inhibitor (SA Fo-Fc omit map contoured at 2.5σ level) is shown as grey mesh. Representations and color coding scheme same as in Fig. 2, save that the bottom panel depicts comparison of the binding mode of CX-6258 and AMP-PNP to PIM1. Magnesium atoms are depicted as orange spheres.

The structure of PIM1- CX-6258 complex was determined at 2.44 Å resolution and the inhibitor is clearly defined by its electron density save for the methyl-1,4-diazepane moiety which points away of the binding site and into the solvent. The interaction is driven by an oxindole ring, which locates at the binding site of adenine was it the ATP substrate (PDB ID 1XR1^7^, Fig. 4A,B). Nevertheless, the interactions guiding the binding of the oxindole and adenine are dissimilar. The oxygen within the inhibitor oxindole forms a hydrogen bond with the sidechain amide of Lys67 and is further stabilized by carbonyl-π interaction with the sidechain of Phe49. The oxindole secondary amine forms a water mediated contact with the main chain amide of Asp186. Further, the water network anchors the inhibitor at the sidechain of Glu89. The oxindole moiety is additionally stabilized by hydrophobic interactions contributed by the sidechains of Val52, Ala65, Ile104, Leu120, Leu174 and Ile185. The chlorine substituent contributes general hydrophobic interactions at the hinge region. The binding of furan moiety is mediated by hydrophobic interaction with the sidechain of Phe49 and the backbone of the G-loop. Interestingly, the loop, partially disordered in a number of PIM1 structures is stabilized here by the said interactions and thus well defined by the electron density. The phenyl moiety contributes hydrophobic interactions with the sidechains of Leu44 and Val126 and C_β_ of Asp128. Additional carbonyl-π interaction connects the inhibitor at the backbone of Leu44. Further linker contributes a weak interaction with the sidechain of Asp131. The methyl-1,4-diazepane moiety points into the solvent and does not contribute significant interactions with the protein. Consequently, it is characterized by poor electron density and high temperature factors.

The binding mode of oxindole moiety of the inhibitor roughly mimics the binding of adenine as demonstrated by comparison of the structure of CX-6258 and AMP-PNP substrate mimetic (Fig. 4B), although oxindole is shifted by approximately 2 Å in the direction of the A-loop. The furan moiety locates at the site comparable to ribose, but the similarities end at this point. The phenyl and diazepane moieties of the inhibitor point in the direction of the hinge region, whereas the phosphate moieties of AMP-PNP are directed towards the A-loop and contribute an extended interaction with the enzyme. Considering the interactions, adenine ring anchors at Glu121 mainchain carbonyl with N6 mediated hydrogen bond, while CX-6258 does not contribute any hydrogen bonds in this region. The position of the carbonyl oxygen within oxindole moiety is almost identical to that of an α phosphonyl oxygen and both anchor at Lys67 sidechain. The binding of furan and ribose moieties is guided by comparable interactions with the G-loop. Further than that, CX-6258 extends towards the hinge region and outside the molecule, while the G-loop folds where the phosphonate moiety is located in the AMP-PNP containing structure. In this regard, the conformation of the G-loop in CX-6258 complex resembles that found in apo-PIM1 structure, an unusual feature for a ligand bound PIM1.

## Discussion

Multiple PIM1 kinase inhibitors have been reported in the recent years. Despite the diversity in their chemical structure, they all target the ATP binding pocket situated between the hinge region, the A-loop and the G-loop. In the majority of examples a cyclic scaffold occupies the binding site of adenine, though the particular interactions are diverse and usually do not directly repeat those of the substrate.

Despite the vast chemical space covered by known inhibitors of protein kinases these molecules functionally fall into three major groups based on the interaction features and the conformation of DFG motif within the activation loop of the kinase^31^. Type I inhibitors bind kinases in their catalytically competent state (usually induced by phosphorylation). Type II inhibitors recognize inactive kinase conformation. Type III inhibitors bind at allosteric sites^32^. PIM kinases are constitutively active (even in the absence of phosphorylation) and in this regard all the inhibitors are of type I while further subdivision is PIM specific.

PIM inhibitors are divided into three major classes, according to the mechanistic mode of interaction with the kinase. ATP-mimetics reproduce the interaction of ATP molecule with Glu121 in the hinge region of the enzyme. Non-ATP mimetic inhibitors are characterized by their interaction with Lys67, a residue critical for sustaining the catalytically active state of PIM1 by forming a salt bridge with Glu89. The salt bridge, together with closed lobe conformation and well-structured activation segment characterize active state of protein kinases in general. Thus, PIM1 does not require phosphorylation for catalytic activity^7,33^. The third class of inhibitors benefits of both the above interactions^5^. Following staurosporine, a prototype inhibitor of a wide range of protein kinases^34^, the majority of reported PIM1 inhibitors belong to ATP-mimetics. Unfortunately for drug development, this class is characterized by a broad kinase inhibitory profile. Because non-ATP mimetics do not interact with the residues at the hinge region – which are most conservative among kinases, but rather with residues at the opposite side of the binding pocket - which are more characteristic for particular kinases^5^, these inhibitors tend to be more specific. Some research suggests that interactions at the hinge region of PIM1 do not contribute significant affinity^35^ though such conclusions should be treated with caution as these are most likely compound specific. Concerning the nuisance of off-target effects of candidate clinical kinase inhibitors, it is of interest that all the compounds characterized in this study are ATP-competitive, non-ATP mimetics. Neither of tested inhibitors anchors at Glu121 while they all form hydrogen bonds with the sidechain amine of Lys67. Additionally, all three compounds interact with Asp186 mainchain carbonyl, a residue from the DFG motif, which together with Lys67 is responsible for maintaining the constitutively active conformation of PIM1 by formation of a number of polar interactions stabilizing the activation loop.

Binding of small molecule inhibitors to PIM1 kinase may also associate with conformational changes within the residues flanking the ATP-binding site. Qian *et al*.^7^ demonstrated that ATP binding to PIM1 follows an induced fit mechanism. A significant conformational change affects primarily the residues within the G-loop (residues 44–52). This region is known to be responsible for determination of specificity, selectivity and affinity of protein kinase inhibitors^35^. Comparison of structures of apo-PIM1 and the kinase in complex with non-hydrolysable analogue of ATP (AMP-PNP) demonstrates an extensive shift within the loop with Phe49 being displaced by as much as around 7 Å. Induction of a similar displacement of the G-loop by ligands was documented for multiple previously described PIM1 inhibitors^36^. Interestingly, the displacement is not relevant to CX-6258, where the conformation of the loop is comparable to that found in the apo-structure. In complex containing CX-4945 the loop is partially unstructured, though some weak electron density suggests a conformation comparable to the CX-6258 containing structure is possible. The conformation of Ro-3306 at the active site is sterically incompatible with the apo-conformation of the loop. It is thus clearly displaced, however its disposition in the complex structure is not defined by the electron density. It was previously suggested that for energetic reasons inhibitors which do not impose any significant conformational changes within the glycine-rich loop of PIM1 kinase may be kinetically favored as opposed to the inhibitors which binding is connected with energetically expensive induced fit within the G-loop^35^. However, such conclusion is difficult to directly evaluate experimentally.

Importantly, the structures provided in this study suggest multiple avenues for further compound development. Alleviating the off-target effects or potentiating dual activities, converting the CX-4945 (CK2α) and Ro-3306 (CDK1) into PIM1 specific inhibitors and potentiating the Flt-3 inhibition by CX-6258 emerge as possible options.

CX-4945 (silmitasertib) is developed in clinics as casein kinase 2 (CK2α) inhibitor^9^, but demonstrates an off-target effect at PIM1. It is not surprising, given almost identical binding modes of CX-4945 at the ATP pocket of CK2α and PIM1, as demonstrated here, but the structures also explain better affinity of the compound towards CK2α. What distinguishes the two binding modes, is an additional, water mediated interaction at His160 of CK2α. Further, CK2α provides a hydrogen bond in the hinge region, while the binding pocket is more spacious in PIM1 and no such interaction is observed. Because of a wider pocket in PIM1 it is difficult to suggest modifications which would decrease PIM1 affinity without affecting CK2α interaction. In fact, multiple derivatives of CX-4945 have shown inhibition of PIM1^10^. It is, however, possible to suggest modifications which would direct the compounds specifically at PIM1. Extending CX-4945 in the direction of the hinge could provide additional favorable interactions with PIM1 which would at the same time most probably abolish CK2α affinity due to steric hindrance. Also because of the differences in the pocket size, it is essential that the carboxylic acid moiety is fixed at position 4 for CK2α, while PIM1 tolerates its transfer to position 3^37^ (Fig. 1A), most likely associated in slightly different orientation of the aromatic core of the compound at the binding pocket.

Ro-3306 has been developed as CDK1 inhibitor, but demonstrates an off-target effect at PIM1. However, our study reveals that it is a different geometric isomer of Ro-3306 which binds to CDK and PIM1. Therefore, isomer separation would facilitate exact targeting of each enzyme separately. The position of quinoline moiety is comparable in both structures. Less spacious binding pocket around the hinge region provides additional interactions with the quinoline and increased affinity of Ro-3306 and CDK1 compared to PIM1. Extension of this moiety could provide favorable contacts with PIM1 to increase the inhibitor affinity. Also, modification of thiophene moiety could afford more favorable interactions with the G-loop.

Prior to our crystal structure, a theoretical binding model was proposed by Haddach *et al*.^12^ to explain the affinity of CX-6258 precursor, a compound identified by high-throughput screening for PIM1 inhibitors. They suggested that the hydrogen bond donor in an oxindole ring anchors at the sidechain of Asp186. The convex of a C-shaped inhibitor would point towards the hinge and the inhibitor would locate parallel to the hinge backbone and anchor with its other end at Asp128 and Glu171 via water mediated interactions. Our results do not support such binding model. The convex of a C-shaped inhibitor is directed opposite to the hinge region. The inhibitor anchors at Lys67 sidechain with the oxygen atom of the oxindole. At the other side it anchors directly at the sidechain of Asp131. Compared to our structure, the precursor lacks the diazepane and chloride moieties, but it seems unlikely that such differences would result in a completely different binding mode, especially that the diazepane moiety points outside the binding pocket and does not provide any significant interactions.

CX-6258 is a selective PIM1, PIM2 and PIM3 kinase inhibitor. It also beneficially inhibits Flt-3 kinase although with relatively weak affinity. Analysis of the binding mode revealed by our structure in the context of structural information on Flt-3 suggests that introduction of a hydrogen bond donor into the phenyl moiety could provide favorable interactions at the hinge region of Flt-3 without affecting PIM affinity. This could deliver a potent pan-PIM/Flt3-inhibitor with therapeutic benefit.

Overall, we obtained crystal structures of PIM1 kinase with three chemically diverse inhibitors providing solid basis for further compound optimization and development.
